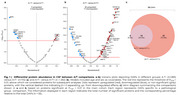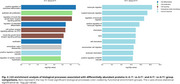# Aging senescence‐related proteins indicates Alzheimer’s disease progression

**DOI:** 10.1002/alz70861_108999

**Published:** 2025-12-23

**Authors:** Lorenzo Celia Ferreira, Rodrigo Sebben Paes, Gabriela Mantovani Baldasso, Christian Limberger, Eduardo R. Zimmer

**Affiliations:** ^1^ Universidade Federal do Rio Grande do Sul, Porto Alegre, Rio Grande do Sul Brazil; ^2^ Brain Institute of Rio Grande do Sul (InsCer), PUCRS, Porto Alegre, Rio Grande do Sul Brazil; ^3^ McGill Centre for Studies in Aging, Montreal, QC Canada

## Abstract

**Background:**

Cellular senescence—a state of permanent growth arrest without cell death—is associated with aging and Alzheimer's disease (AD). The accumulation of senescent cells in the brain contributes to tissue dysfunction and promotes neurodegenerative processes implicated in AD pathogenesis, suggesting a potential role in disease progression. Using the validated SenMayo senescence‐associated biomarker panel, we analyzed molecular signatures and biological processes in cerebrospinal fluid (CSF) to investigate their interplay with AD pathology.

**Method:**

We analyzed 680 ADNI individuals at baseline, divided in A/T status (A‐T‐, A+T‐ and A+T+), representing AD biological progression. Amyloid and Tau positivity were defined by CSF pTau‐181/Aβ₄₂ ratio (>0.025) and pTau‐181 levels (>27 pg/ml), respectively. Differential abundance of 106 senescence‐related proteins from the SenMayo panel was assessed using age‐ and sex‐adjusted linear models. Proteins were considered differentially abundant if P_FDR_ < 0.01. Then, Gene Ontology (GO) enrichment of Biological Processes was performed using clusterProfiler. All analyses were performed in R.

**Result:**

We identified 14 differentially abundant proteins (DAPs ), 5 upregulated and 9 downregulated, in the A+T‐ compared to A‐T‐ group (Figure 1a) and 55 DAPs, 38 upregulated and 17 downregulated, in A+T+ (Figure 1b) if compared to A+T‐ group. All DAPs present in A+T‐ are also present in A+T+ individuals (Figure 1c). Enrichment analysis for Biological Processes revealed that cell differentiation, proliferation, and signaling were predominantly associated with the A+T‐, whereas the A+T+ was linked to immune response (Figure 2).

**Conclusion:**

Our findings reveal a distinct senescence‐associated proteomic signature across the biological course of AD, with a marked increase in DAPs linked to senescence as AD pathology progresses. These changes may reflect underlying associations between cellular senescence and the amyloid and tau phases of the disease, highlighting the potential of this biomarker panel as a valuable framework for understanding the pathological transition across AD progression.